# Palliative Care in Shanghai Communities: Needs, Influencing Factors and Preferences Among Older Adults with Chronic Diseases

**DOI:** 10.3390/healthcare13233048

**Published:** 2025-11-25

**Authors:** Jiameng Li, Jiangjiang He, Zhenqing Tang, Limei Jing, Lin Zhu

**Affiliations:** 1 School of Psychology, Shanghai Normal University, 100 Guilin Road, Xuhui District, Shanghai 200234, China; jiamengli@zju.edu.cn; 2 School of Tourism, Shanghai Normal University, 100 Guilin Road, Xuhui District, Shanghai 200234, China; 3 Shanghai Health Development Research Center (Shanghai Medical Information Center), 1477 Beijing West Road, Jingan District, Shanghai 200040, China; hejiangjiang@shdrc.org (J.H.); tangzhenqing@shdrc.org (Z.T.); 4 School of Public Health, Shanghai Jiao Tong University School of Medicine, 227 South Chongqing Road, Huangpu District, Shanghai 200025, China

**Keywords:** palliative care, older adults, chronic diseases, Shanghai communities

## Abstract

**Background:** Palliative care plays a vital role in enhancing the quality of life of older adults with chronic diseases, while service coverage in China remains limited. Shanghai faces increasing demand due to its rapidly aging population and high prevalence of chronic diseases. However, little is known about the needs, influencing factors, and care preferences of community-dwelling older adults. This study aimed to assess palliative care needs in this population and to identify associated determinants and care preferences. **Methods:** A cross-sectional survey was conducted among community-dwelling older adults with chronic diseases in Shanghai. Data on sociodemographic information, palliative care needs and preferences were collected. Descriptive analyses were used to summarize participant characteristics, chi-square tests assessed group differences, and logistic regression models examined predictors of palliative care needs. **Results:** A total of 611 older adults participated, of whom 6.5% were identified as having palliative care needs. Dependency in activities of daily living (*p* = 0.024) and poorer self-rated health (*p* = 0.001) were significantly associated with increased palliative care needs. Participants demonstrated a greater preference for comfort-oriented care for themselves than for family members. Gender differences were observed in care setting preferences: females were more likely to favor palliative care institutions, while males tended to choose non-palliative care wards in general hospitals. **Conclusions:** Even in Shanghai, where palliative care services are relatively advanced compared with other regions of China, awareness and utilization remain low among older adults with chronic conditions. Functional decline and negative self-rated health are associated with palliative care needs, underscoring the importance of incorporating functional and health assessments into community health services to identify eligible individuals earlier. The observed discrepancy between self- and family-oriented preferences, alongside gendered differences in care setting choices, highlights the need for culturally sensitive communication and tailored service provision. Expanding community-based palliative care and increasing public education may help align services with the needs and preferences of older adults in China.

## 1. Background

### 1.1. Palliative Care and Its Coverage

With global population aging, the prevalence of chronic diseases is rising sharply [1,2]. Older adults often live with multiple chronic diseases such as cardiovascular disease, chronic respiratory disease or diabetes [3]. These illnesses are frequently accompanied by pain, fatigue, sleep problems, and reduced mobility, in addition to psychological and social distress. These multidimensional challenges significantly impair quality of life and increase the need for comprehensive care. Palliative care has emerged as a critical approach for addressing these challenges. According to the World Health Organization, it aims to improve the quality of life of patients facing life-threatening illness, as well as their families, by providing relief from pain, symptom distress, and psychosocial suffering [4]. Beyond symptom management, palliative care supports communication, shared decision-making, and preparation for the end of life [5]. Evidence from high-income countries suggests that early integration of palliative care not only enhances quality of life and satisfaction with care but may also reduce unnecessary hospitalizations and healthcare costs [6,7].

Despite its importance, palliative care coverage worldwide remains uneven. In Europe, countries like the UK and Germany have well-established palliative care systems integrated into national health policies, while in Asia, countries such as South Korea and Japan have made significant strides through national hospice and palliative care initiatives [8,9,10]. The Global Atlas of Palliative Care reports that outside North America, Europe, and Australia, access to quality palliative care continues to be minimal, even though 76% of the need is in low-and-middle income countries [11].

According to the 2025 Global Ranking of Palliative Care Development, China is classified at the “Established” level, with a Global Development Score (GDS) of 2615. It ranks 11th out of 41 countries and territories in the Asia-Pacific region [12]. Most palliative care units are located in cancer hospitals or tertiary general hospitals, focusing primarily on advanced cancer patients [13], while community-based services for older adults with non-malignant chronic diseases remain limited [14]. Cultural barriers also play a role. Deeply rooted traditions, such as the Confucian ethic of filial piety and the belief that “life and death are preordained,” shape attitudes toward end-of-life care [15,16,17]. As a result, palliative care is often perceived as a form of “giving up,” and decisions to initiate it may impose psychological and social pressure on families. These factors have contributed to the slow integration of palliative care into China’s mainstream healthcare system, despite the growing need.

### 1.2. Palliative Care Policy in China and Shanghai

Recognizing these challenges, the Chinese government has introduced a series of initiatives to promote palliative care. In February 2017, the National Health Commission issued the Basic Standards and Management Regulations for Palliative Care Centers [13], which provided the first formal policy framework for the development of services. Later that year, national pilot programs were launched, followed by a second expansion in 2019 that included Shanghai as one of the pilot sites [18].

Shanghai has played a pioneering role in this national initiative. Since 2012, the municipal government has repeatedly emphasized the development of palliative and hospice services in its work reports, practical projects, and long-term planning [19]. By 2024, substantial progress had been achieved in Shanghai, with 261 medical institutions providing palliative care services, including 248 community health centers and 13 general hospitals [20]. Community health service centers in Shanghai are primary care facilities providing basic medical services, chronic disease management, and public health education to registered residents. These centers serve as the frontline of Shanghai’s public healthcare system. These developments show a strong policy commitment to expanding access to palliative care and integrating it into primary healthcare systems.

However, despite these efforts, the actual utilization of palliative care services in Shanghai remains limited. Many older adults and their families are unaware of the existence or the meaning of palliative care [21]. This gap between policy ambition and real-world uptake suggests the need for a better understanding of community-level needs, influencing factors, and preferences among older adults and their families.

### 1.3. The Necessity of an Assessment on Palliative Care Needs in Shanghai

Shanghai is at the forefront of population aging in China. By the end of 2023, the city’s registered population aged 60 and above had reached 5.68 million, accounting for 37.4% of the total population in Shanghai [22]. This proportion is considerably higher than the national average and places Shanghai in a stage of pronounced aging. Chronic diseases are highly prevalent among this population, contributing to functional decline, reduced quality of life, and increasing demand for long-term care.

While Shanghai has invested significant resources in chronic disease prevention and management, there is still limited research on palliative care needs outside of oncology. Most existing studies in China have focused on hospitalized cancer patients, with an emphasis on clinical symptom control [23]. Few studies have examined the needs of community-dwelling older adults with multiple chronic conditions [14], despite their large and growing numbers. Moreover, cultural and family dynamics play a particularly important role in shaping care preferences in China, yet little is known about how these factors influence decision-making among community residents in Shanghai. Addressing this gap is crucial for guiding service planning, optimizing resource allocation, and ensuring that palliative care models meet the real-world needs of older adults and their families.

Therefore, the current study aimed to explore palliative care in Shanghai communities. Guided by Andersen’s Behavioral Model of Health Service Use [24], which examines how predisposing, enabling, and need factors influence health service utilization, the objectives were to: (1) Assess the prevalence of palliative care needs among community-dwelling older adults with chronic diseases using the Palliative Care Screening Tool (PCST), (2) identify sociodemographic and health-related factors associated with these needs, and (3) explore preferences for end-of-life care, including treatment choices and preferred care settings, and examine potential gender differences in these preferences.

## 2. Methods

### 2.1. Participants

A cross-sectional study was conducted from February to March 2025 using stratified sampling. Community health service centers were selected from districts in Shanghai, including suburban areas (Pudong New District, Jiading District), outer suburban areas (Qingpu District, Fengxian District), and central urban areas (Xuhui District, Putuo District). In each district, 1–3 community health service centers were chosen based on the size of the local older adult population. The study included 11 community health service centers, with a final list of participating centers detailed in Table 1.

Quota sampling was employed, with approximately 60 older adults with chronic diseases randomly selected from each community. A total of 660 questionnaires were distributed and collected, of which 49 were excluded based on the predefined criteria, with a valid response rate of 92.6%. Eligible participants were those aged ≥ 60 years with a diagnosis of at least one chronic disease (e.g., cardiovascular or cerebrovascular disease, chronic respiratory disease, diabetes, or cancer). Exclusion criteria included impaired consciousness, mental disorders, cognitive impairments, or hearing impairments that prevented participation. For participants unable to respond clearly to certain questions, family members were allowed to assist.

### 2.2. Measures

The draft questionnaire was piloted among 10 older patients from the target group, who were also asked to provide specific feedback about clarity and appropriateness. The final questionnaire comprised the following components:

#### 2.2.1. General Information Questionnaire

This section included sociodemographic and health-related information. Sociodemographic variables included age, sex, highest education attained, marital status, number of children, living arrangements, economic status, and primary caregiver. Health-related information covered the number and types of diagnosed chronic diseases, disease duration, surgical history, activities of daily living, and self-rated health status. Self-rated health was assessed using the first item of the Short-Form Health Survey (SF-36) [25].

#### 2.2.2. Palliative Care Screening Tool (PCST)

The PCST was developed in 2018 to assess palliative care needs among older adults with chronic diseases in community settings [26]. The tool consists of 22 items across three dimensions: physical symptoms, emotional concerns, and goals of care. The physical symptoms dimension includes items such as pain, fatigue, and sleep difficulties. The emotional concerns dimension includes items such as anxiety, uncontrollable worry, and depression. The goals of care dimension consist of two parts: (1) perceptions of and needs for long-term care, and (2) advance medical directives. Except for advance medical directives items, all items are rated on a three-point scale (“never” = 0, “sometimes” = 1, “always” = 2). For long-term care planning items, the response options are “yes” (scored as 1), “no” (scored as 2), and “uncertain” (scored as 2). Higher total scores indicate greater palliative care needs. Participants were considered to have palliative care needs if they selected “always” on two or more items in at least two dimensions [27]. The PCST was cross-culturally adapted and validated in Chinese populations, showing good reliability (Cronbach’s α = 0.824) [14]. Internal consistency for the PCST in our sample was α = 0.896.

#### 2.2.3. Palliative Care Preference Scale

This scale included six items across three domains: (1) willingness to receive life-sustaining treatments (LST), (2) preferences regarding palliative care options and timing, and (3) preferences regarding palliative care services and care settings [28,29]. As the items did not have predetermined or standardized answers, no total score was calculated.

### 2.3. Data Collection

First, ethical approval was obtained from the Ethics Committee of Shanghai Health Development Research Center (protocol number 2021004). Second, the research protocol was submitted to the hospital nursing management department and the heads of each clinical department for review and approval. Two postgraduate students in health management were trained as investigators to administer the questionnaire survey. They were assisted by nursing staff at each community health service center. The study content was explained in detail to participants, and any health-related questions were addressed. All collected patient information was kept strictly confidential.

### 2.4. Statistical Analyses

Statistical analyses were conducted using SPSS 25.0. Descriptive statistics were used to summarize sociodemographic characteristics and health-related factors, and chi-square tests were used to examine group differences. The outcome variable was palliative care needs (a dichotomous variable). Based on the results of univariate analyses, variables with statistical significance were included as independent variables in a binary logistic regression model, with sociodemographic factors entered as control variables and other health-related factors as key explanatory variables. Multicollinearity among independent variables was assessed using variance inflation factors (VIFs), all of which were below 2.0, indicating no significant multicollinearity [30]. Additionally, descriptive statistics and chi-square tests were used to analyze gender differences in palliative care preferences. A *p*-value < 0.05 was considered statistically significant for all analyses [31].

## 3. Results

General sociodemographic and other background information of 611 valid samples are presented in Table 2. The mean age of participants was 71.09 (SD = 0.32) years, with more female (61.4%) than male (38.6%). Most participants had a junior high school education or below and were living with a spouse or children, reflecting typical family arrangements in urban Chinese communities. Most participants (74.6%) were primarily cared for by their spouse or children, reflecting the dominant family-based caregiving model in China. While nearly all respondents were covered by medical insurance, the majority reported modest household incomes (<6000 RMB per month), which is low relative to the city’s cost of living (compared to monthly per capita disposable income of 7364 RMB). Hypertension was the most prevalent chronic condition, and many participants had long-standing health problems, with almost half living with chronic diseases for over five years. A total of 260 participants (42.5%) were taking three or more medications. Approximately half of the sample rated their overall health as fair or poor, suggesting substantial disease burden despite preserved daily functioning in most cases (86.7% performed basic activities of daily living independently).

Table 2 presents the prevalence of palliative care needs across different subgroups. Significant variations were observed among some populations. Within the age range of 60–89 years, older participants were more likely to report palliative care needs. The absence of children was also associated with higher needs, underscoring the role of family support in sustaining care at home. Conversely, those with only one child were less likely to report such needs, possibly reflecting adequate but limited caregiving resources. Participants with medical insurance, multiple chronic conditions, or taking several medications reported a higher prevalence of palliative care needs. This suggests that health complexity and treatment intensity are key indicators of unmet supportive requirements. Furthermore, participants who were completely dependent in activities of daily living, as well as those with poorer self-rated health, were more likely to report palliative care needs.

Of the 611 elderly patients with chronic diseases in Shanghai communities, the detailed palliative care needs across the three dimensions are presented in Table 3. Among physical symptoms, the most prevalent were sleep difficulties (104, 17%), difficulty standing or walking (60, 9.8%), and physical pain (59, 9.7%). Among emotional concerns, the most frequently reported were reduced interest in usual activities (35, 5.7%), concern of being a burden to family or friends (30, 4.9%), and feeling down or depressed (25, 4.1%). Regarding goals of care, the most prevalent were the need for access to more medical providers (120, 19.6%), and the need for more information about community health resources (105, 17.2%).

Table 4 shows the adjusted logistic regression results for factors associated with palliative care needs. After controlling for age, number of children, living arrangement, health insurance status, and primary caregiver, two predictors remained significant. Older adults who were completely dependent in activities of daily living had nearly seven times higher odds of reporting palliative care needs [OR = 6.98, 95% CI (1.29, 37.7), *p* = 0.024], compared with those who were independent. Similarly, poorer self-rated health was strongly associated with increased palliative care needs, with participants rating their health as fair [OR = 6.86, 95% CI (2.27, 20.79), *p* = 0.001], or poor [OR = 10.56, 95% CI (2.50, 44.59), *p* = 0.001] showing greater likelihood of need than those reporting good health. These findings indicate that both functional dependency and perceived health status are associated with palliative care demand. Incorporating these simple indicators into community health screening could help identify at-risk older adults early and guide timely supportive interventions.

Table 5 presents palliative care preferences of 40 older adults with chronic diseases identified as positive by the PCST. Among them, 32.5% expressed willingness to receive life-sustaining treatments. If diagnosed with a terminal illness, more than half preferred to receive appropriate palliative care, which may not prolong life but can provide greater comfort. However, when considering family members with a terminal illness, only 40% preferred that their family members receive such care, whereas 47.5% preferred their family members to prolong life as much as possible, even in the presence of pain, discomfort, or suffering. If they or a family member were diagnosed with a terminal illness, 52.5% would be willing to discuss palliative care during treatment or upon its completion. Regarding the preferred setting for end-of-life care, 67.5% identified home as most appropriate. A significant gender difference was observed: 31.8% of females preferred palliative care institutions or wards, while only 5.6% of males preferred the same. Males showed a preference for general hospitals, whereas females did not. At the end of life, 82.5% reported a preference for symptom management and comfort care to relieve suffering.

## 4. Discussion

This study contributes to the emerging literature on palliative care among community-dwelling older adults with chronic diseases in Shanghai. Applying Andersen’s Behavioral Model, our findings reveal how predisposing (e.g., age, gender), enabling (e.g., family support, insurance), and need factors (e.g., functional status, self-rated health) influence palliative care needs and preferences in the Shanghai context. Overall, only 6.5% were identified by the PCST as having palliative care needs, with physical symptoms, particularly sleep difficulties, mobility limitations, and pain, being more frequently reported than emotional concerns. Around one fifth reported a desire for more communication with healthcare professionals or for more information about community health resources. When compared internationally, this prevalence is lower than reports from both Chinese and international studies [4,26,27,32], which may be attributed to differences in measurement tools, disease severity, and populations. 

Dependency in activities of daily living and poorer self-rated health were independently associated with palliative care needs. Among those identified as positive by the PCST, only one third were willing to receive life-sustaining treatments. While 52.5% preferred comfort-oriented palliative care for themselves in the event of a terminal illness, fewer (40%) expressed the same preference for family members, often favoring life-prolonging care for family members despite potential suffering. A notable gender difference was observed in the preferred setting for end-of-life care: females were more likely to favor palliative care institutions, whereas males preferred nursing homes and non-palliative care wards in general hospitals.

Several contextual factors may explain the relatively low prevalence of identified needs in our sample. The participants were primarily urban residents in Shanghai, where the healthcare system is relatively well developed and accessible. Shanghai ranks among the Chinese cities with the highest life expectancy—over 83 years, significantly exceeding the national average (approximately 76 years) and close to the world’s highest of 83.7 in Japan and 83.4 in Switzerland [33,34]. Furthermore, 86.7% of participants in this study remain functionally independent, which may delay the emergence of needs requiring palliative care. These contextual factors may help explain the relatively low prevalence observed in our sample, though they do not negate the importance of addressing the symptom burden among those identified as having palliative care needs. The physical symptoms, particularly sleep difficulties, mobility limitations, and pain, were the most commonly reported areas where early palliative approaches could play a role in improving quality of life, even before individuals meet criteria for formal palliative care needs.

Our findings revealed that dependency in activities of daily living and poorer self-rated health were significantly associated with palliative care needs. These results are consistent with previous studies indicating that functional decline and perceived poor health are strong predictors of palliative care needs [35,36]. Poor self-care ability or negative self-rated health may reflect a decline in physical functioning, greater disease burden, or increased reliance on others for daily activities [37,38]. These conditions often translate into more complex care needs across physiological, psychological, and social domains. In the Chinese context, where family caregiving continues to be the primary source of support, functional dependence not only reflects the burden of disease but also imposes a significant burden on families. This dual strain, on both the individual and the caregiving system, explains why functional decline and poor perceived health are strong indicators of palliative care needs.

Among participants identified as having palliative care needs, only one-third expressed willingness to receive life-sustaining treatments, while more than half preferred comfort-oriented care in the event of terminal illness. Interestingly, participants were more likely to endorse comfort care for themselves than for family members. This discrepancy reflects the deeply rooted cultural norms of filial piety and family-centered decision-making in China [5], where prolonging a family member’s life, even at the cost of suffering, may be seen as a moral responsibility. Similar findings have been observed in other studies, suggesting that end-of-life preferences are strongly shaped by cultural and familial values rather than individual autonomy alone [39]. This pattern aligns with findings from other Asian contexts, such as Japan, where family-centered decision-making often overrides individual preferences [40]. Moreover, a notable gender difference was observed in preferences for the setting of end-of-life care. While similar proportions of males and females preferred home-based care, females were more likely to favor palliative care institutions, whereas males tended to choose general hospital wards that do not provide palliative services. The findings align with prior research indicating that females have more favorable attitudes toward palliative care [36]. One possible explanation is that females, who often take on caregiving roles themselves, may better recognize the value of specialized palliative care in relieving symptom burden and providing holistic support. In contrast, males may place greater trust in conventional hospital-based care focused on life maintenance. These patterns highlight the complexity of gendered perceptions of care and the need to tailor service provision to address different expectations.

This study has several limitations. First, it was conducted in selected communities in Shanghai and may not be generalizable to other regions in China, especially rural areas with fewer resources. The findings may not reflect the entire Shanghai older adult population due to potential selection bias. Second, the cross-sectional design does not allow for causal inferences between health status and palliative care needs. Third, reliance on self-reported preferences may not fully reflect actual decision-making behaviors at the end of life. Fourth, the absence of qualitative data limits our understanding of the underlying reasons for the observed preferences. Future studies should adopt longitudinal designs, incorporate the perspectives of family caregivers and healthcare providers, and include comparative analyses across different regions of China to better align individual needs with culturally appropriate, family-centered care.

## 5. Policy Implications

Our findings have several practical implications for developing community-based palliative care in China.

Even in Shanghai, one of the most developed regions in the country, most older adults have little awareness of what palliative care entails. Raising awareness and fostering health literacy around palliative care should be a priority, not only in Shanghai but especially in less resourced regions of Mainland China [41]. Community health authorities could launch public education campaigns to demystify palliative care, emphasizing its role in improving quality of life rather than being synonymous with “giving up.”

Dependency in activities of daily living and poorer self-rated health are strong indicators of palliative care needs. These factors could be incorporated into routine community health assessments to help identify older adults who may benefit from early supportive interventions. Moreover, a simplified screening tool based on these key predictors (ADL dependency and self-rated health) could be developed and integrated into the electronic health records at community health centers to facilitate routine screening. In parallel, support should be extended to family caregivers, who are the primary source of care in China and may be affected by patients’ declining function [15,42]. Specifically, training modules for general practitioners should be developed to enhance their skills in initiating conversations about end-of-life preferences, managing common symptoms, and understanding the principles of palliative care.

The discrepancy between preferences for oneself and for family members underscores the cultural complexity of end-of-life decision-making in China, where filial piety and family-centered values can lead to prioritizing life-prolongation for relatives, even at the cost of suffering. Policies and professional training should therefore encourage open and culturally sensitive communication that balances individual wishes with family perspectives [43,44]. In addition, the national legislative process regarding living will system should be accelerated. The observed gender differences in institutional preferences highlight the importance of tailoring service provision [45]. Females’ greater inclination toward palliative care institutions suggests an openness to specialized supportive services, whereas males’ preference for general hospital wards reflects a reliance on conventional medical models. Policymakers and providers should recognize these gendered patterns and ensure that service options are diverse, accessible, and responsive to different expectations. For instance, service navigation and counseling could be provided to help older adults and their families choose care settings that best align with their values and needs.

## 6. Conclusions

In summary, this study provides new insights into the palliative care needs and preferences of community-dwelling older adults with chronic diseases in Shanghai. These findings highlight the urgency of promoting public awareness, integrating functional and health assessments into community health practice, and developing services that are culturally appropriate and responsive to diverse expectations. The specific factors identified (functional dependency and self-rated health) offer a pragmatic basis for developing community-level screening protocols. The documented preferences for care, and the tensions between self and family choices, provide a clear mandate for training healthcare workers in communication and for designing supportive interventions for families. Strengthening palliative care in Shanghai can serve as a model for broader national efforts, but sustained attention is required to ensure that older adults across China can access and benefit from high-quality, person- and family-centered palliative care.

## Figures and Tables

**Table 1:** List of participating community health service centers.

Name of District	Name of Community Health Service Center
**Suburban area**	
Pudong New District (3)	NMT community health service center
	JY community health service center
	SQ community health service center
Jiading District (2)	NX community health service center
	AT community health service center
**Outer suburban area**	
Qingpu District (1)	XJ community health service center
Fengxian District (1)	XD community health service center
**Central urban area**	
Xuhui District (2)	FL community health service center
	TL community health service center
Putuo District (2)	CF community health service center
	YC community health service center

**Table 2:** A univariate analysis of general information and palliative care needs of elderly patients with chronic diseases in the community, N (%).

	Total, (N = 611)	No Need, (N = 571)	Have Need, (N = 40)	χ^2^	*p*
**Age**				14.619	0.002
60–69	293 (47.9)	280 (95.6)	13 (4.4)		
70–79	216 (35.3)	203 (94.0)	13 (6.0)		
80–89	91 (14.8)	77 (84.6)	14 (15.4)		
≥90	11 (1.8)	11 (100.0)	0 (0.0)		
**Sex**				0.734	0.392
Male	236 (38.6)	218 (92.4)	18 (7.6)		
Female	375 (61.3)	353 (94.1)	22 (5.9)		
**Highest education attained**				0.653	0.957
Primary school or below	155 (25.3)	143 (92.3)	12 (7.7)		
Middle school	237 (38.7)	223 (94.1)	14 (5.9)		
High/occupational school	150 (24.5)	140 (93.3)	10 (6.7)		
College	39 (6.3)	37 (94.9)	2 (5.1)		
Bachelor or above	30 (4.9)	28 (93.3)	2 (6.7)		
**Marital status**				5.719	0.126
Married	513 (84)	482 (94)	31 (6.0)		
Widowed	84 (13.7)	76 (90.5)	8 (9.5)		
Divorced	11 (1.8)	11 (100)	0		
Unmarried/others	3 (0.5)	2 (66.7)	1 (33.3)		
**Number of children**				12.159	0.016
0	9 (1.4)	7 (77.8)	2 (22.2)		
1	357 (58.4)	343 (96.1)	14 (3.9)		
2	183 (29.9)	165 (90.2)	18 (9.8)		
3	47 (7.6)	42 (89.4)	5 (10.6)		
4	15 (2.4)	14 (93.3)	1 (6.7)		
**Living arrangement**				16.836	0.002
Living alone	69 (11.2)	64 (92.8)	5 (7.2)		
With spouse	402 (65.7)	378 (94.0)	24 (5.9)		
With children	110 (18.8)	106 (96.3)	4 (3.7)		
With relatives	7 (1.1)	6 (85.7)	1 (14.3)		
Nursing homes	23 (3.7)	17 (73.9)	6 (26.1)		
**Have health insurance**				4.489	0.034
Yes	553 (90.5)	513 (92.8)	40 (7.2)		
No	58 (9.4)	58 (100)	0		
**Personal monthly income (RMB)**				1.629	0.443
2000–6000	480 (78.5)	447 (93.1)	33 (6.9)		
6000–10,000	109 (17.8)	102 (93.6)	7 (6.4)		
10,000–30,000	22 (3.6)	22 (100)	0		
**Primary caregiver**				14.641	0.012
Self-care	121 (19.8)	117 (96.7)	4 (3.3)		
Spouse	327 (53.5)	304 (93)	23 (7)		
Children	129 (21.1)	122 (94.6)	7 (5.4)		
Other relatives	2 (0.3)	2 (100)	0		
Domestic helper	6 (0.9)	6 (100)	0		
Community care workers	26 (4.2)	20 (76.9)	6 (23.1)		
**Number of chronic diseases**				14.146	0.003
1	292 (47.8)	279 (95.5)	13 (4.5)		
2	177 (28.4)	169 (95.5)	8 (4.5)		
3	88 (14.4)	76 (86.4)	12 (13.6)		
≥4	54 (8.8)	47 (87.0)	7 (13.0)		
**Type of chronic diseases**				0.574	0.449
Non-cancer chronic diseases	568 (92.9)	532 (93.6)	36 (6.3)		
Malignant tumors	43 (7.0)	39 (90.6)	4 (9.3)		
**Duration of illness (year)**				6.771	0.080
1–3	263 (43.0)	245 (93.2)	18 (6.8)		
3–5	51 (8.3)	49 (96.0)	2 (4.0)		
5–10	86 (14.0)	85 (98.8)	1 (1.2)		
>10	211 (34.5)	192 (91)	19 (9)		
**History of surgery in the past 3 years**				1.242	0.265
Yes	86 (14.1)	78 (90.6)	8 (9.4)		
No	525 (85.9)	493 (93.9)	32 (6.1)		
**Number of different medications used**				17.372	0.001
0	93 (15.2)	92 (99.0)	1 (1.0)		
1	123 (20.1)	120 (98.0)	3 (2.0)		
2	135 (22.0)	128 (94.8)	7 (5.1)		
≥3	260 (42.5)	231 (88.8)	29 (11.1)		
**Activities of daily living (ADL)**				46.297	<0.001
Independent	530 (86.7)	501 (94.5)	29 (5.5)		
Partially dependent	55 (9.0)	54 (98.2)	1 (1.8)		
Totally dependent	26 (4.2)	16 (61.5)	10 (38.5)		
**Self-rated health**				44.704	<0.001
Excellent/very good/good	314 (51.4)	310 (98.7)	4 (1.3)		
Fair	260 (42.5)	234 (90.0)	26 (10.0)		
Poor	37 (6.0)	27 (73.0)	10 (27.0)		

**Table 3:** The current status of palliative care needs for elderly patients with chronic diseases in the community, N (%).

	No	Sometimes	Always
**Physical symptoms**			
Pain	338 (55.3)	214 (35)	59 (9.7)
Tired and fatigued	316 (51.7)	248 (40.6)	47 (7.7)
Difficulty standing or walking	396 (64.8)	155 (25.4)	60 (9.8)
Difficulty sleeping	297 (48.6)	210 (34.4)	104 (17.0)
Shortness of breath	507 (83)	89 (14.6)	15 (2.5)
Poor appetite or weight loss	468 (76.5)	123 (20.1)	20 (3.2)
**Emotional concerns**			
Nervous or anxious	382 (62.5)	211 (34.5)	18 (2.9)
Uncontrollable worry	433 (70.9)	161 (26.4)	17 (2.8)
Little interest in usual activities	413 (67.6)	163 (26.7)	35 (5.7)
Feeling down or depressed	412 (67.4)	174 (28.5)	25 (4.1)
Hopelessness	516 (84.5)	82 (13.4)	13 (2.1)
Concern of being a burden to family or friends	420 (68.7)	161 (26.4)	30 (4.9)
Feeling like no one to talk to	468 (76.6)	124 (20.3)	19 (3.1)
Having conflicts with friends or family	490 (80.2)	106 (17.3)	15 (2.5)
**Goals of care**			
Feeling overwhelmed about any medical treatment	444 (72.7)	152 (24.9)	15 (2.4)
Confused about medical care	473 (77.4)	121 (19.8)	17 (2.8)
Uncomfortable to discuss health-related issues	461 (75.5)	132 (21.6)	18 (2.9)
Need access to more medical providers	268 (43.9)	223 (36.5)	120 (19.6)
Need more information about community health resources	270 (44.2)	236 (38.6)	105 (17.2)
	**Yes**	**No**	**Unclear**
Have you considered how to be cared for when your illness advance/as you age?	288 (47.1)	275 (45.0)	48 (7.9)
Have you talked with anyone about how you want to be cared for?	235 (38.5)	330 (54.0)	46 (7.5)
If YES, do you have a document that indicates what your wishes and who will make decisions for you?	211 (34.5)	350 (57.3)	50 (8.2)

**Table 4:** Logistic regression analysis of palliative care needs for elderly patients with chronic disease.

	B	SE	*p*	OR (95% CI)
Age	0.03	0.26	0.909	1.03 (0.62–1.72)
Number of children	0.07	0.26	0.787	1.07 (0.65–1.77)
Living arrangement	0.11	0.16	0.465	1.12 (0.83–1.52)
Have health insurance	17.98	4845.6	0.997	>1000 (0.00–∞)
Primary caregiver	−0.21	0.21	0.317	0.81 (0.53–1.23)
**Number of chronic diseases**				
1	Ref			
2	−0.69	0.51	0.176	0.50 (0.18–1.36)
3	0.23	0.54	0.673	1.25 (0.44–3.58)
≥4	−0.42	0.63	0.504	0.66 (0.19–2.25)
0	Ref			
1	1.08	1.21	0.37	2.95 (0.28–31.53)
2	1.69	1.14	0.136	5.44 (0.59–50.23)
≥3	2.01	1.11	0.072	7.44 (0.84–66.08)
**Activities of daily living (ADL)**				
Independent	Ref			
Partially dependent	−1.97	1.06	0.064	0.14 (0.02–1.12)
Totally dependent	1.94	0.86	0.024	6.98 (1.29–37.7)
**Self-rated health**				
Excellent/very good/good	Ref			
Fair	1.93	0.57	0.001	6.86 (2.27–20.79)
Poor	2.36	0.74	0.001	10.56 (2.50–44.59)

**Table 5:** Palliative care preferences among community-dwelling older adults with chronic diseases screened positive by PCST.

	Total (40)	Male (18)	Female (22)	χ^2^	*p*
**1. Willingness to receive life-sustaining treatments**	13 (32.5)	6 (33.3)	7 (31.8)	0.01	0.92
**2. Care preference for yourself in terminal illness**				3.77	0.15
(1) prolong life regardless of discomfort	14 (35.0)	7 (38.9)	7 (31.8)		
(2) prioritize comfort through palliative care	21 (52.5)	7 (38.9)	14 (63.6)		
(3) combine life-prolonging with palliative care	5 (12.5)	4 (22.2)	1 (4.5)		
**3. Care preference for family members in terminal illness**				0.86	0.65
(1) Prolong life regardless of discomfort	19 (47.5)	9 (50)	10 (45.5)		
(2) Prioritize comfort through palliative care	16 (40.0)	6 (33.3)	10 (45.5)		
(3) Combine life-prolonging with palliative care	5 (12.5)	3 (16.7)	2 (9.1)		
**4. Timing of willingness to discuss palliative care**				2.43	0.66
(1) At the time of diagnosis	10 (25.0)	5 (27.8)	5 (22.7)		
(2) During the course of treatment	17 (42.5)	6 (33.3)	11 (50)		
(3) Upon completion of treatment	4 (10.0)	3 (16.7)	1 (4.5)		
(4) When around the end of life	6 (15.0)	3 (16.7)	3 (13.6)		
(5) Not willing at any time	3 (7.5)	1 (5.6)	2 (9.1)		
**5. Preferred care setting for terminally ill patients**				10.3	0.036
(1) At home, cared for by family members	16 (40.0)	6 (33.3)	10 (45.5)		
(2) At home, cared for by professional caregivers	11 (27.5)	6 (33.3)	5 (22.7)		
(3) Palliative care institutions or wards	8 (20.0)	1 (5.6)	7 (31.8)		
(4) Nursing homes or long-term care institutions	2 (5.0)	2 (11.1)	0		
(5) General hospital (non-palliative care ward)	3 (7.5)	3 (16.7)	0		
**6. Preferred end-of-life services**				2.8	0.83
(1) Symptom management and comfort care	33 (82.5)	14 (77.8)	19 (86.4)		
(2) Psychological counseling and help	4 (10.0)	2 (11.1)	2 (9.1)		
(3) Support from family and social resources	3 (7.5)	2 (11.1)	1 (4.5)		

## Data Availability

The data presented in this study are available on request from the corresponding author due to ethical reasons.

## References

[1] Xi J.-Y., Lin X., Hao Y.-T. (2022). Measurement and projection of the burden of disease attributable to population aging in 188 countries, 1990–2050: A population-based study. J. Glob. Health.

[2] Karthika M., Abraham J., Kodali P.B., Mathews E. (2023). Emerging Trends of Chronic Diseases and Their Care Among Older Persons Globally. Handbook of Aging, Health and Public Policy.

[3] Chen Q.-F., Ni C., Jiang Y., Chen L., Liao H., Gao J., Qin X., Pan S., Luan X., Wu Y. (2025). Global burden of disease and its risk factors for adults aged 70 and older across 204 countries and territories: A comprehensive analysis of the Global Burden of Disease Study 2021. BMC Geriatr..

[4] Wang X., Mo Y., Yuan Y., Zhou Y., Chen Y., Sheng J., Liu J. (2023). Exploring the influencing factors of unmet palliative care needs in Chinese patients with end-stage renal disease undergoing maintenance hemodialysis: A cross-sectional study. BMC Palliat. Care.

[5] He F.X., Geng X., Johnson A. (2021). The experience of palliative care among older Chinese people in nursing homes: A scoping review. Int. J. Nurs. Stud..

[6] Wichmann A.B. (2020). Decreased costs and retained QoL due to the ‘PACE Steps to Success’ intervention in LTCFs: Cost-effectiveness analysis of a randomized controlled trial. BMC Med..

[7] Seow H., Barbera L.C., McGrail K., Burge F., Guthrie D.M., Lawson B., Chan K.K.W., Peacock S.J., Sutradhar R. (2022). Effect of Early Palliative Care on End-of-Life Health Care Costs: A Population-Based, Propensity Score–Matched Cohort Study. JCO Oncol. Pract..

[8] Cerullo G., Figueiredo T., Coelho C., Campos C.S., Videira-Silva A., Carrilho J., Midão L., Costa E. (2024). Palliative Care in the Ageing European Population: A Cross-Country Comparison. Int. J. Environ. Res. Public Health.

[9] Kim H.S., Kim B.H., Ferrell B.R., Coyle N., Paice J.A. (2015). Palliative care in South Korea. Oxford Textbook of Palliative Nursing.

[10] Miyashita M., Morita T., Hirai K. (2008). Evaluation of end-of-life cancer care from the perspective of bereaved family members: The Japanese experience. J. Clin. Oncol..

[11] World Health Organization (2020). Worldwide Hospice Palliative Care Alliance. Global Atlas of Palliative Care.

[12] Tripodoro V.A., Fidalgo J.F.L., Pons J.J., Connor S., Garralda E., Bastos F., Montero Á., Llamas L.M., Béjar A.C., Suárez D. (2025). First-Ever Global Ranking of Palliative Care: 2025 World Map Under the New WHO Framework. J. Pain Symptom Manag..

[13] Li X., Wang X.S., Huang H., Liu M., Wu Y., Qiu J., Zhang B., Cui L., Hui D. (2023). National survey on the availability of oncology palliative care services at tertiary general and cancer hospitals in China. BMC Palliat. Care.

[14] Zhang K., Peng Y., Liu L., Gu C. (2023). The status quo and influencing factors of palliative care needs of the community elderly with chronic diseases. Chin. Nurs. Manag..

[15] Zhang Y., Zhang S., Liu C., Chen X., Ding Y., Guan C., Hu X. (2023). Caregiver burden among family caregivers of patients with advanced cancer in a palliative context: A mixed-method study. J. Clin. Nurs..

[16] Ng W.I., Che S.L., Li X., Zhu M. (2024). Association of filial attitude, filial behavior and death literacy: Implications for development of death system in Guangdong-Hong Kong-Macao Greater Bay Area of China. BMC Public Health.

[17] Zheng H., Li R., Ma H., Cheng Q., Zhang Y., Gong Y., Luo L. (2025). A multicenter cross-sectional study on nurses’ attitudes toward end-of-life care and influencing factors: A latent profile analysis. BMC Nurs..

[18] Ning X. (2019). Hospice and palliative care research in mainland China: Current status and future direction. Palliat. Med..

[19] Du Q. (2015). Bringing Dignity to Death. Global Times.

[20] Cai W. (2024). Compassionate Hospice Care Available Across Shanghai. City News Service Shanghai.

[21] Che S.L., Li X., Zhu M., Ng W.I. (2023). The Death Literacy Index: Translation, cultural adaptation, and validation of the Chinese version. Front. Public Health.

[22] Zheng Z. (2025). Shanghai Ramps Up Elderly Care Amid Aging Population. China Daily.

[23] Zhao X.-X., Cui M., Geng Y.-H., Yang Y.-L. (2019). A systematic review and meta-analysis of randomized controlled trials of palliative care for pain among Chinese adults with cancer. BMC Palliat. Care.

[24] Andersen R.M. (1995). Revisiting the behavioral model and access to medical care: Does it matter?. J. Health Soc. Behav..

[25] Li L., Wang H.M., Shen Y. (2003). Chinese SF-36 Health Survey: Translation, cultural adaptation, validation, and normalisation. J. Epidemiol. Community Health.

[26] Ghesquiere A., Gardner D.S., McAfee C., Kenien C., Capezuti E., Kozlov E., Sirey J.A., Reid M.C. (2018). Development of a Community-Based Palliative Care Screening Tool for Underserved Older Adults With Chronic Illnesses. Am. J. Hosp. Palliat. Care.

[27] Kozlov E., Cai A., Sirey J.A., Ghesquiere A., Reid M.C. (2018). Identifying Palliative Care Needs Among Older Adults in Nonclinical Settings. Am. J. Hosp. Palliat. Med..

[28] Gauthier D.M., Froman R.D. (2001). Preferences for care near the end of life: Scale development and validation. Res. Nurs. Health.

[29] Perry L.M., Hoerger M., Malhotra S., Gerhart J.I., Mohile S., Duberstein P.R. (2020). Development and Validation of the Palliative Care Attitudes Scale (PCAS-9): A Measure of Patient Attitudes Toward Palliative Care. J. Pain. Symptom Manag..

[30] Conteh E.N., Bangura F.S. (2024). The Effect of Training and Development Practices on Employees’ Performance in Commercial Banks in Sierra Leone: Case of Union Trust Bank (UTB). Int. J. Sci. Bus..

[31] Das D., Das T. (2023). The “P”-Value: The Primary Alphabet of Research Revisited. Int. J. Prev. Med..

[32] Wang L., Li Y., Zhao R., Li J., Gong X., Li H., Chi Y. (2024). Influencing factors of home hospice care needs of the older adults with chronic diseases at the end of life in China: A cross-sectional study. Front. Public Health.

[33] Zhou W. (2017). Life Expectancy in Shanghai Tops 83 Years. China Daily.

[34] Chen H., Hao L., Yang C., Yan B., Sun Q., Sun L., Chen H., Chen Y. (2018). Understanding the rapid increase in life expectancy in shanghai, China: A population-based retrospective analysis. BMC Public Health.

[35] Osse B.H., Vernooij-Dassen M.J., de Vree B.P., Schadé E., Grol R.P. (2000). Assessment of the need for palliative care as perceived by individual cancer patients and their families: A review of instruments for improving patient participation in palliative care. Cancer.

[36] Lu X., Liu J. (2022). Factors Influencing Public Awareness of and Attitudes Toward Palliative Care: A Cross-Sectional Analysis of the 2018 HINTS Data. Front. Public Health.

[37] Lee J., Abdel-Kader K., Yabes J.G., Cai M., Chang H.-H., Jhamb M. (2021). Association of Self-Rated Health with Functional Limitations in Patients With CKD. Kidney Med..

[38] Mavaddat N., Valderas J.M., Van Der Linde R., Khaw K.T., Kinmonth A.L. (2014). Association of self-rated health with multimorbidity, chronic disease and psychosocial factors in a large middle-aged and older cohort from general practice: A cross-sectional study. BMC Fam. Pract..

[39] Lee M.C., Hinderer K.A., Alexander C.S. (2018). What Matters Most at the End-of-Life for Chinese Americans?. Gerontol. Geriatr. Med..

[40] Fukui S., Yoshiuchi K., Fujita J., Sawai M., Watanabe M. (2011). Japanese people’s preference for place of end-of-life care and death: A population-based nationwide survey. J. Pain. Symptom Manag..

[41] Lin H., Huang Y., Wang Y. (2025). Facilitators and barriers to palliative care delivery in rural China: A qualitative study of the perceptions and experiences of rural healthcare professionals. BMC Palliat. Care.

[42] Zhang X., Xu T., Qin Y., Wang M., Li Z., Song J., Tang Q., Wang Z., Xu L., Wu L. (2023). Exploring the needs and coping strategies of family caregivers taking care of dying patients at home: A field study. BMC Palliat. Care.

[43] Zuo T., Yeap S.Y., Lu G. (2025). Navigating the delicate balance of autonomy and harmony: A case study on the cultural adaptation of palliative care interventions in China. BMC Palliat. Care.

[44] Shih Y.-A., Wang C., Ding J., Zhou Y., Lu Q. (2025). Cultural and ethical barriers to implementing end-of-life advance care planning among oncology nursing professionals: A content analysis of open-ended questions. BMC Med. Ethics.

[45] Narayanan D., Chandrasekaran A.S., S E.A.R., Vyas N. (2025). Gender disparities in end-of-life care: A scoping review of patient, caregiver and care provider perspectives in low-and middle-income countries. BMC Palliat. Care.

